# Author Correction: OmpK36-mediated Carbapenem resistance attenuates ST258 *Klebsiella pneumoniae* in vivo

**DOI:** 10.1038/s41467-023-37549-y

**Published:** 2023-04-06

**Authors:** Joshua L. C. Wong, Maria Romano, Louise E. Kerry, Hok-Sau Kwong, Wen-Wen Low, Stephen J. Brett, Abigail Clements, Konstantinos Beis, Gad Frankel

**Affiliations:** 1 Centre for Molecular Bacteriology and Infection, Department of Life Sciences, Imperial College London, London, UK; 2 Department of Surgery and Cancer, Section of Anaesthetics, Pain Medicine and Intensive Care, Imperial College London, London, UK; 3 Department of Life Sciences, Imperial College London, London, UK; 4 Rutherford Appleton Laboratory, Research Complex at Harwell, Didcot, Oxfordshire UK

Correction to: *Nature Communications* 10.1038/s41467-019-11756-y, published online 02 September 2019

The original version of this Article contained errors in Table 1. The correct version of the 2nd row (‘ICC8002’) states ‘OmpK35_WT_’ instead of the original, incorrect ‘OmpK35_ST258_’, and ‘OmpK36_ST258_’ instead of the original, incorrect ‘OmpK36_WT_’. The correct version of the 3rd row (‘ICC8003’) states ‘OmpK35_ST258_’ instead of the original, incorrect ‘OmpK35_WT_’, and ‘OmpK36_WT_’ instead of the original, incorrect ‘OmpK36_ST258_’. This has been corrected in both the PDF and HTML versions of the Article.

