# Supplementary material for: OmpK36-mediated Carbapenem resistance attenuates ST258 Klebsiella pneumoniae in vivo
Source: Nat Commun. 2019 Sep 2;10:3957. doi: 10.1038/s41467-019-11756-y (PMC6718652; doi:10.1038/s41467-019-11756-y)
Supplement: Supplementary file 1 — Supplementary Information [file 41467_2019_11756_MOESM1_ESM.pdf]

Supplementary information

OmpK36-mediated Carbapenem resistance attenuates ST258 *Klebsiella pneumoniae in vivo*

Wong et al.

Supplementary Tables

Supplementary Table 1. Strains used in this study.

**Strain Number**	**Strain name**	**Resistance**	**Notes**	**Reference**
S1	ICC8001	Rifampicin	ATCC43816 serially passaged *in vitro* on Rifampicin containing LB plates to 100mcg/ml followed by two passages in BALB/c mice.	This study
S2	CC118λpir	NA	Maintains the R6K oriR of pSEVA612S used in this work for mutagenesis	Lab collection
S3	E Coli pRK2013	Kanamycin	Helper strain in triparental mating	Figurski and Helinski 1979
S4	KPST258	Carbapenems	Reference MLST258 *K. pneumoniae* strain for this study containing KPC-2 on a pKpQIL-like background.	Public Health England
S5	KPOXA-48	Carbapenems	*K. pneumoniae* strain containing OXA-48 on a pOXA48a-like background.	Public Health Enlgand
S6	DH5α	NA	E. Coli strain used in this study.	Lab collection
S7	E. Coli BL21(DE3)omp 8	Kanamycin	Porin deficient strain used in this study.	Prilipov et al 1998

Supplementary Table 2. Vectors used in this study.

**Vector (number)**	**Plasmid**	**Resistance**	**Reference**
1	pSEVA612S	Gentamicin	Silva-Rocha et al 2013
2	pACBSR	Streptomycin	Ruano-Gallego et al 2015
3	pSEVA-OmpK35WT	Gentamicin	This study
4	pSEVA-Ompk35ST258	Gentamicin	This study
5	pSEVA-dOmpK36	Gentamicin	This study
6	pSEVA-subOmpK36St258	Gentamicin	This study
7	pSEVA-OmpK36ST258dGD	Gentamicin	This study
8	pSEVA-OmpK36WT	Gentamicin	This study
9	pSEVA-Kp-N2-Lux	Gentamicin	This study
10	pGEmat-lux	Gentamicin	Gift from Luis Angel Fernandez
11	pUltraGFP-Gm	Gentamicin	Gift from Despoina Mavridou
12	pUltraRFP-Gm	Gentamicin	Gift from Despoina Mavridou
13	pSEVA-Kp-N2-GFP	Gentamicin	This study
14	pSEVA-Kp-N2-RFP	Gentamicin	This study
15	pEBMSCHIS	Ampicillin	

Supplementary Table 3. Primer used in this study.

Primer (Number)	Sequence 5' to 3'
1	GGATTACCCTGTTATCCCTAATGAAGCGCAATATTCTGGC
2	TATAGGGATAACAGGGTAATTTAGAACTGGTAAACGATACCC
3	GTATCGTTTACCAGTTCTAAATTACCCTGTTATCCCTATACT
4	GCCAGAATATTGCGCTTCATTAGGGATAACAGGGTAATCCG
5	AGCACGTCTGGACCACCAATGGC
6	CGCCCGACCATTTTTCCATAGAAGTCCAG
7	GCCGACTGATTAGAAGGGTAATC
8	GAGTATACCAGCGAGGTTAAACC
9	GGATTACCCTGTTATCCCTAGGCCTAATTGATTGATTAATAGTCG
10	TTTGTTATGCAGCTTGCAACGTTATTAACCCTCTGTTTGTTATATG
11	ACAAACAGAGGGTTAATAACGTTGCAAGCTGCATAACAAAAAGG
12	TATAGGGATAACAGGGTAATAGCCCCACAGGTTGACCAGC
13	GCTGGTCAACCTGTGGGGCTATTACCCTGTTATCCCTATACTG
14	ATTAATCAATCAATTAGGCCTAGGGATAACAGGGTAATCCG
15	ACAAACAGAGGGTTAATAACATGAAAGTTAAAGTACTGTCCCT
16	TTTGTTATGCAGCTTGCAACTTAGAACTGGTAAACCAGGC
17	GGCGACACCTACGGTTCT
18	GCCGAATTCCGGCAGAAC
19	TTTGTTATGCAGCTTGCAACTTAGAACTGGTAAACCAGGC
20	ACAAACAGAGGGTTAATAACATGAAAGTTAAAGTACTGTCCCT
21	CGGCGACGGCGACACCTACGGTTC
22	CCGAATTCCGGCAGAACG
23	TTCGGATCCAGGACAAGCGTATTGAG
24	CTGGGTACCCCATGGCCCTTTGCAAGATAGGATGC
25	ATGGGTACCCTCGAGGCTGCCGACGGCGGCCCTTTT
26	ACCGAATTCCAATACCCTGGTAGTTC
27	CTGCCATGGTTATCAAAAAGAGTATTGGC

28	GATCTCGAGACTAGTTCAACTATCAAACG
29	GCTGCCGACGGCGGCCCT
30	CCTTTGCAAGATAGGATGCTTTAC
31	ATCCTATCTTGCAAAGGCGATAGAGTATTGACTTCG
32	AGGGCCGCCGTCGGCAGCCGCGAAGTAATCTTTTCG
33	GGTCAGGATGCGTCTATCG
34	CCTGAGTCAGTTTGTCATC
35	ACCAACAACGGTCGTACCGCC
36	AGCGCCTTCGCCGCTGAC
37	CCTGCAGTCCGCTGCTAACGGCG
38	AAGTTGTCAGAACCGTAG
39	TTCATGCGCTCTATCGGCGATA
40	GCATAGTCATTTGCCGTGCCAT
41	GATTATGGTAATGAGGACATTTCGGGC
42	CATATCCATATTCATCGCAAAAAACCACAC

Supplementary Figures

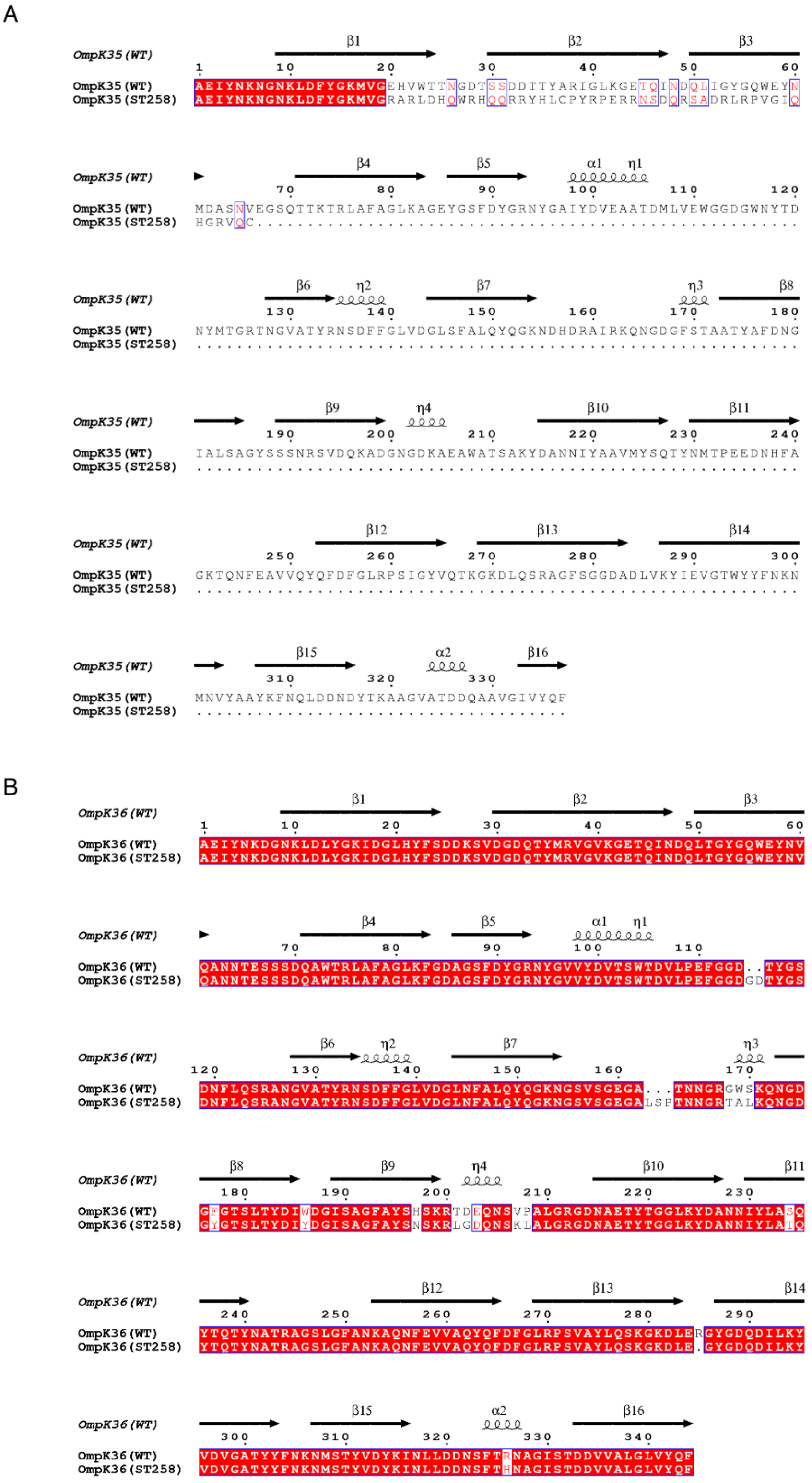

Supplementary Figure 1. Alignment of WT and ST258 OmpK35 **(A)** and OmpK36 **(B).**

A

	IPM	MEM	ETP	AMP	AMC	TZP	CTX	CZA	CAZ	FEP	C_T	ATM
ICC8001	0.25	<=0.060	<=0.125	64	4	8	<=0.125	0.25	0.25	<=0.125	<=0.250	<=0.125
ICC8002	0.25	<=0.060	<=0.125	32	2	8	<=0.125	0.125	0.25	<=0.125	<=0.250	<=0.125
ICC8003	0.25	<=0.060	<=0.125	32	2	8	<=0.125	0.25	0.25	<=0.125	0.5	<=0.125
ICC8004	0.5	<=0.060	0.5	64	4	8	0.25	0.25	0.25	0.25	0.5	0.25
ICC8001 + KPC-2	4	1	8	>64	>64	>64	8	0.25	32	2	8	64
ICC8002 + KPC-2	16	16	>16	>64	>64	>64	32	0.5	16	16	8	64
ICC8003 + KPC-2	8	8	16	>64	>64	>64	8	0.5	32	2	8	>64
ICC8004 + KPC-2	32	32	>16	>64	>64	>64	32	1	32	32	16	>64
ICC8001 + OXA-48	2	0.5	4	>64	>64	>64	0.5	0.25	0.25	<=0.125	1	<=0.125
ICC8002 + OXA-48	8	4	16	>64	>64	>64	2	0.25	0.25	1	1	<=0.125
ICC8003 + OXA-48	2	0.5	8	>64	>64	>64	0.5	0.25	0.25	<=0.125	2	<=0.125
ICC8004 + OXA-48	8	16	>16	>64	>64	>64	8	0.25	0.25	2	2	0.25

B

	CIP	TOB	AMK	GEN	TGC
ICC8001	<=0.125	0.25	1	0.25	1
ICC8002	<=0.125	0.25	<=0.5	0.25	1
ICC8003	<=0.125	0.25	<=0.5	0.25	1
ICC8004	<=0.125	0.5	1	0.25	1
ICC8001 + KPC-2	<=0.125	0.25	1	0.25	1
ICC8002 + KPC-2	<=0.125	0.5	<=0.5	0.25	1
ICC8003 + KPC-2	<=0.125	0.25	<=0.5	0.25	1
ICC8004 + KPC-2	<=0.125	0.25	1	0.25	1
ICC8001 + OXA-48	<=0.125	0.25	1	0.25	1
ICC8002 + OXA-48	<=0.125	0.5	1	0.25	1
ICC8003 + OXA-48	<=0.125	0.5	<=0.5	0.25	1
ICC8004 + OXA-48	<=0.125	0.25	<=0.5	0.25	1

Key

- n=MIC (mg/L) Sensitive
- n=MIC (mg/L) Intermediate
- n=MIC (mg/L) Resistant

Supplementary Figure 2. The impact of $OmpK35_{ST258}$ and $OmpK36_{ST258}$ substitution on resistance to antibiotics used in Gram-negative infection. The MICs of the isogenic KP strains were determined in the absence or presence of the carbapenemases KPC-2 or OXA-48. Individual values are colour coded according to their antibiotic resistance defined by EUCAST breakpoints (Green-sensitive, Orange-intermediate and Red-resistant).

A. Carbapenem resistance requires both porin modification and expression of carbapenemases. Strains remain sensitive to other β-lactam antibiotics (except Ampicillin, in keeping with the genome blaSHV-11) without KPC-2 or OXA-48. **B.** Porin mutation and carbapenemase expression do not impact on the resistance of non-β-lactam antibiotic classes.

Antibiotic Key: IPM Imipenem, MEM Meropenem, ETP Ertapenem, AMP Ampicillin, AMC Amoxicillin/Clavulanate (2:1), TZP Pipericillin/Tazobactam, CTX Cefotaxime, CZA Ceftazidime/Avibactam, CAZ Ceftazidime, FEP Cefipime, C_T Ceftolozone/Tazobactam, ATM Aztreonam, IPM Imipenem, MEM Meropenem, ETP Ertapenem, CIP Ciprofloxicin, TOB Tobramycin, AMK Amikacin, GEN Gentamicin, TGC Tigecycline.

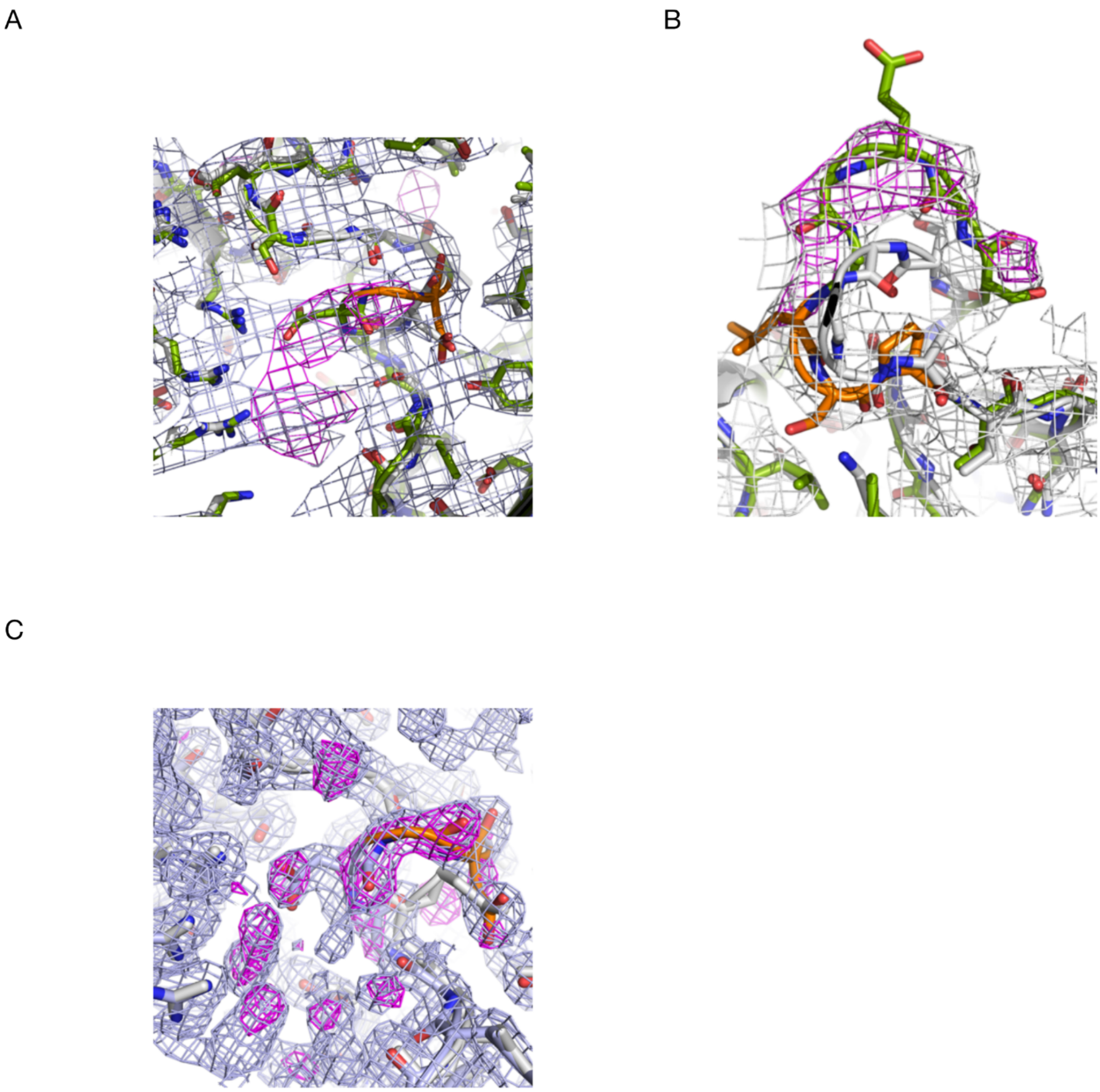

Supplementary Figure 3.

Electron density maps for the OmpK36$_{ST258}$ and OmpK36$_{WT+GD}$ crystal structures.

(A) Positive F_o-F_c electron density can be observed for the conformational change of L3 as a result of the GD insertion in OmpK36$_{ST258}$ structure (green sticks) after molecular replacement with the OmpK36$_{wt}$ (PDB ID: 5O79); the electron density map is shown as magenta mesh contoured at 3 σ. The $2F_o$-F_c electron density map shows good electron density for the surrounding side chains (blue mesh contoured at 1 σ). The GD insertion from the refined

structure is shown for reference (orange sticks) and has not been included in the calculation of this map. The OmpK36$_{wt}$ is shown for reference (grey cartoon).

(B) Positive F_o-F_c electron density (electron density map is shown as magenta mesh contoured at 3 σ) can also be observed for the conformational changes in L4 as a result of the insertions (orange sticks). The insertions from the final refined structure are shown for reference and have not been included in the map calculation.

(C) Similarly, positive F_o-F_c electron density (electron density map is shown as magenta mesh contoured at 3 σ) can be observed for the GD insertion (orange sticks) in L3 of the OmpK36$_{WT+GD}$ structure (blue sticks).

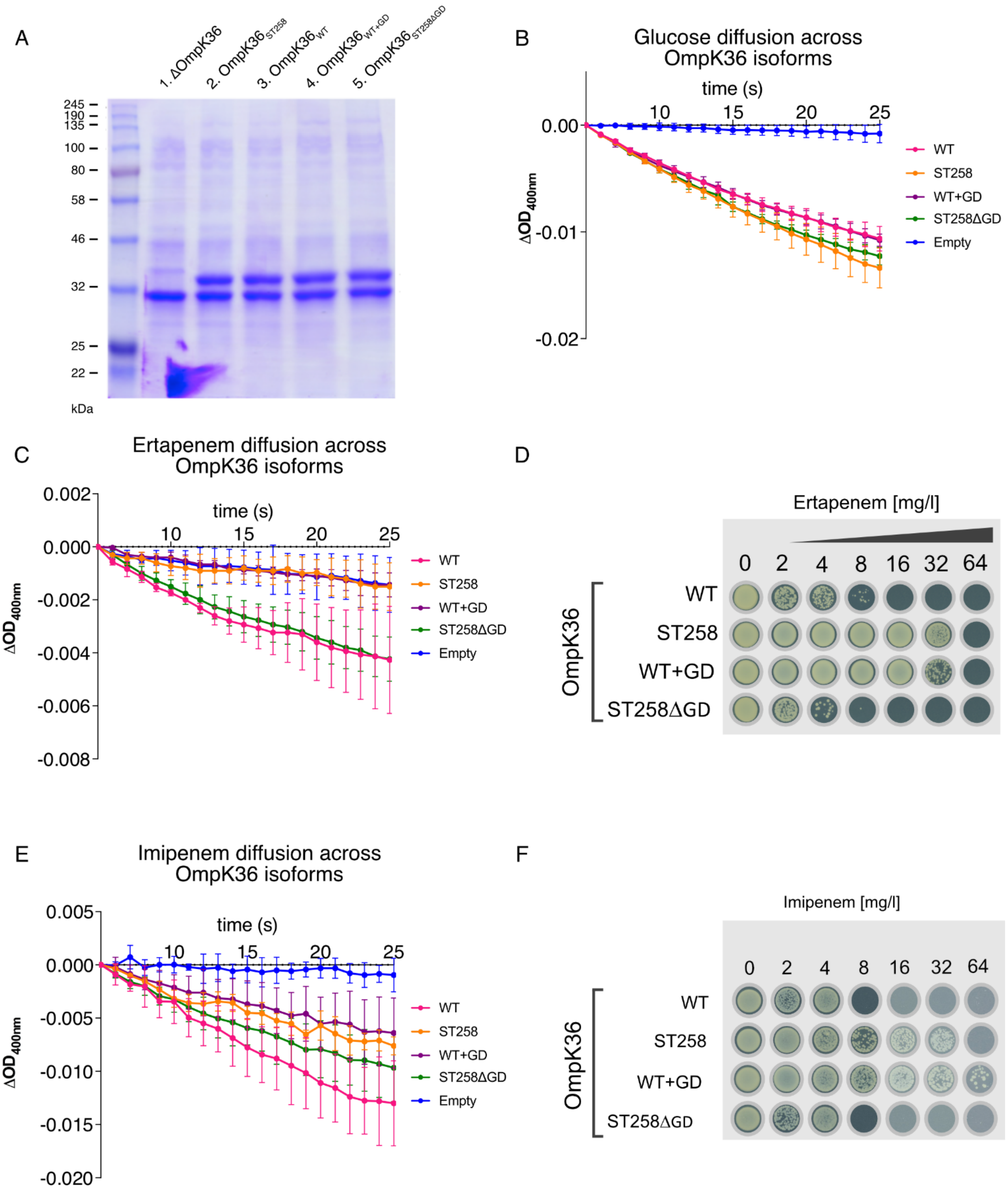

Supplementary Figure 4. The GD insertion in L3 reduces diffusion and mediates resistance to Ertapenem and Imipenem.

A. Outer membrane preparations of the isogenic ICC8003 strains (harboring OmpK35$_{ST258}$) expressing the different OmpK36 isoforms (lanes 2-5). ICC8003 harboring a deletion of

OmpK36 was used as a control (lane 1). All OmpK36 isoforms are expressed in similar levels in outer membrane preparations.

B. Glucose diffuses across all the OmpK36 isoforms but not into control liposomes lacking OmpK36 (Empty). (n=3 per protein isoform, error bars±s.d.).

Diffusion of Ertapenem (475g/mol, **C**) and Imipenem (299g/mol, **E**) is hindered by the GD L3 insertion. (n=3 per protein isoform, error bars±s.d.).

Resistance to Ertapenem (**D**) and Imipenem (**F**) (on $OmpK35_{ST258}$ background in the presence of KPC-2) is mediated by the GD L3 insertion.

A.

Carbohydrate	Molar Mass (g/mol)
Glucose	180.156
Lactose	342.297
Stachyose	666.579

B.

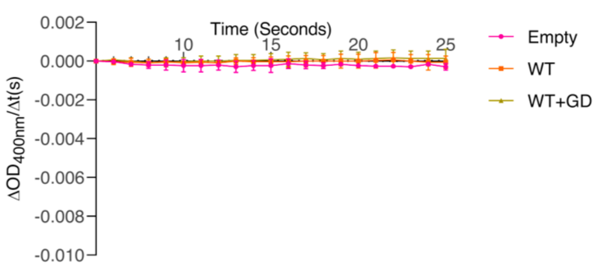

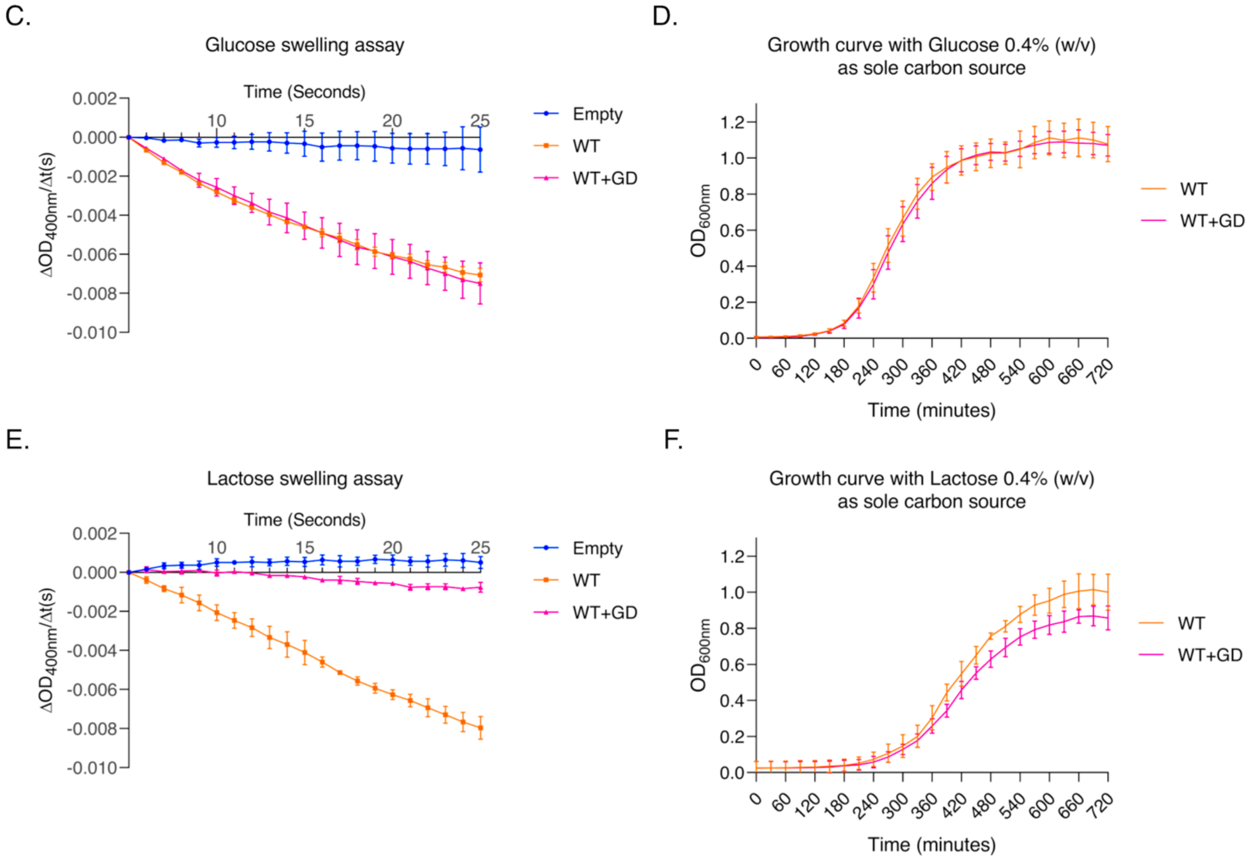

Supplementary Figure 5. The GD insertion in L3 of OmpK36 impairs diffusion of disaccharides and results in an *in vitro* growth impairment.

A. The molecular masses of the three carbohydrates tested in swelling assay.

B. The tetrasaccharide Stachyose is unable to diffuse through either the OmpK36$_{WT}$ or OmpK36$_{WT+GD}$ pore (n=3 repeats, error bars=±s.d.).

C. The monosaccharide glucose diffuses freely through both OmpK36$_{WT}$ and OmpK36$_{WT+GD}$

D. There is no apparent change in growth between isogenic strains expressing either OmpK36 variant in medium containing glucose as the sole carbon source (n=3 repeats, error bars=±s.d.).

E. Diffusion of the disaccharide lactose through $OmpK36_{WT+GD}$ is reduced compared to $OmpK36_{WT.}$ (n=3 repeats, error bars=±s.d.).

F. The isogenic strain expressing $OmpK36_{WT+GD}$ grows slower and attains a lower final OD_{600nm} at stationary phase in medium containing lactose as the sole carbon source (n=3 repeats, error bars=±s.d.).

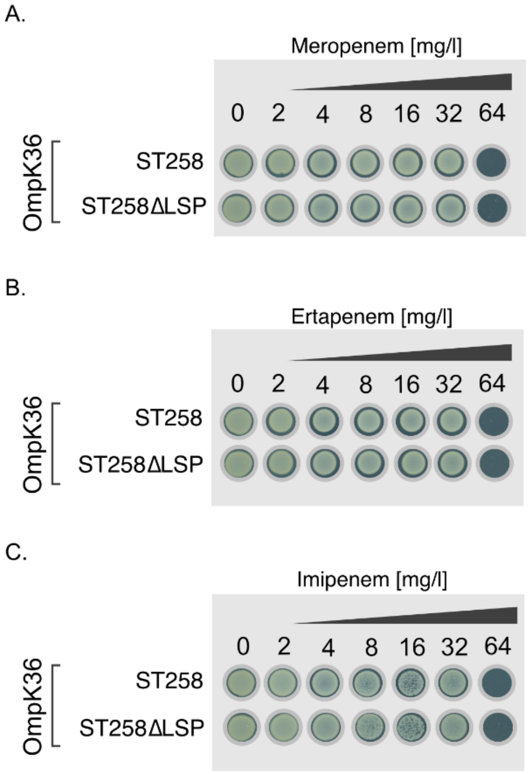

Supplementary Figure 6. The LSP insertion in L4 does not contribute to resistance to Carbapenems.

A. Meropenem, **B.** Ertapenem and **C.** Imipenem resistance is unchanged in isogenic strains expressing $OmpK36_{ST258}$ or $OmpK36_{ST258\Delta LSP}$ (on an $OmpK35_{ST258}$ background) in the presence of KPC-2 carbapenemase.

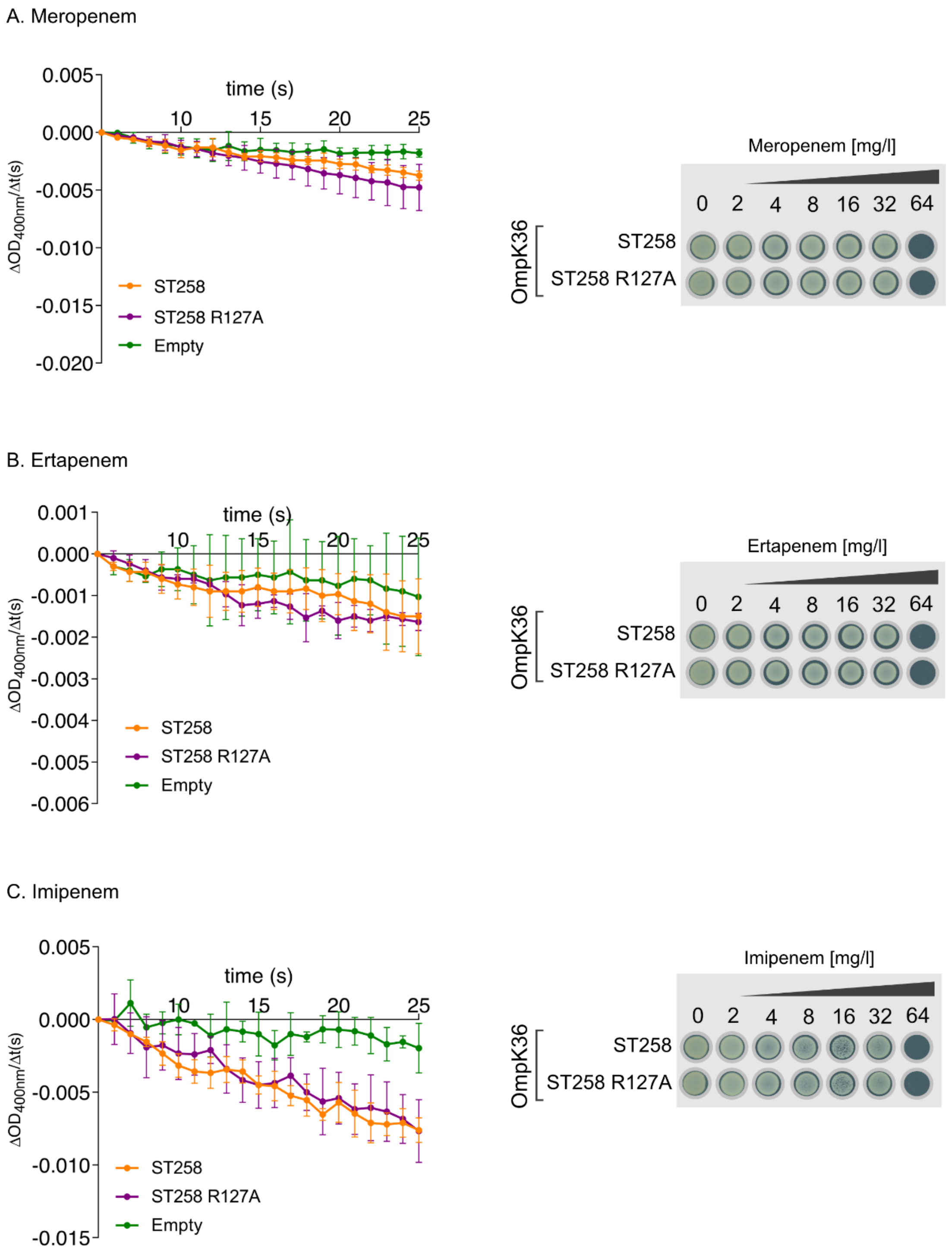

Supplementary Figure 7. The D114-R127 salt-bridge has no role in Carbapenems diffusion or increased resistance.

Disruption of the salt bridge by the R127A mutant does not alter diffusion of Meropenem (**A**), Ertapenem (**B**) or Imipenem (**C**), as measured by liposomal swelling assay(n=3 repeats, error bars=±s.d.)., or result in a change in minimum inhibitory concentration to these antibiotics.

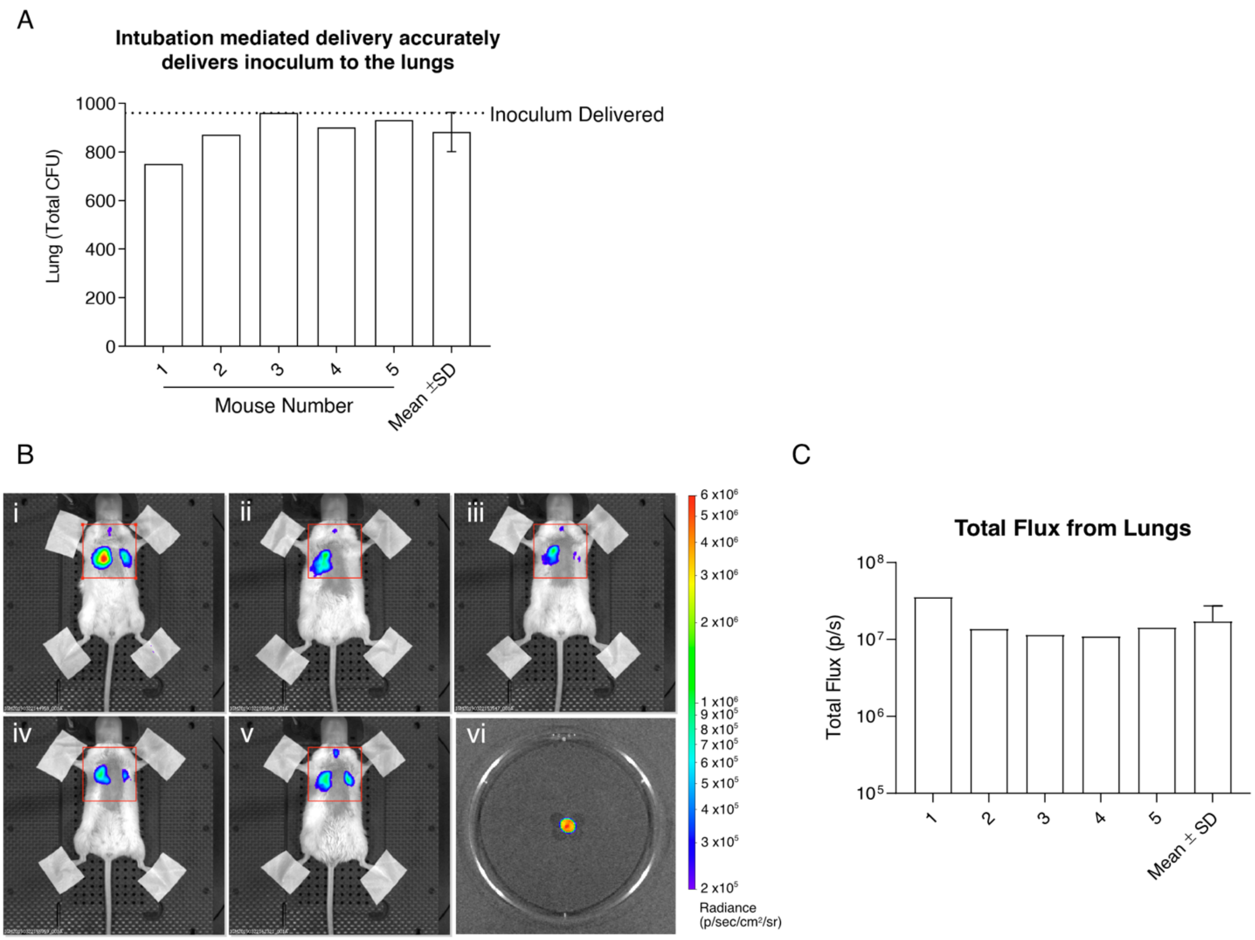

Supplementary Figure 8. *In vivo* imaging of KP in the Ventilator-associated pneumonia model.

A. Validation of the efficiency and accuracy of bacterial delivery directly into the lungs. Mice were intubated with 1000CFU and their lungs were immediately homogenised and bacteria enumerated. This data demonstrates that the initial dose is delivered at high accuracy with the almost entire dose reaching the parenchyma. Error bars±s.d..

B. i-v. Individual mice were intubated and inoculated with bioluminescent ICC8001 (inoculum imaged alone in **vi**) and imaged using IVIS SpectrumCT. The red box in the 2D image indicates the region of interest analysed in Living Image Studio (Caliper Life Sceinces, Massachustes) for total flux (photos/second).

C. Total flux measures in region of interest in each mouse (n=5 mice). Error bars±s.d..

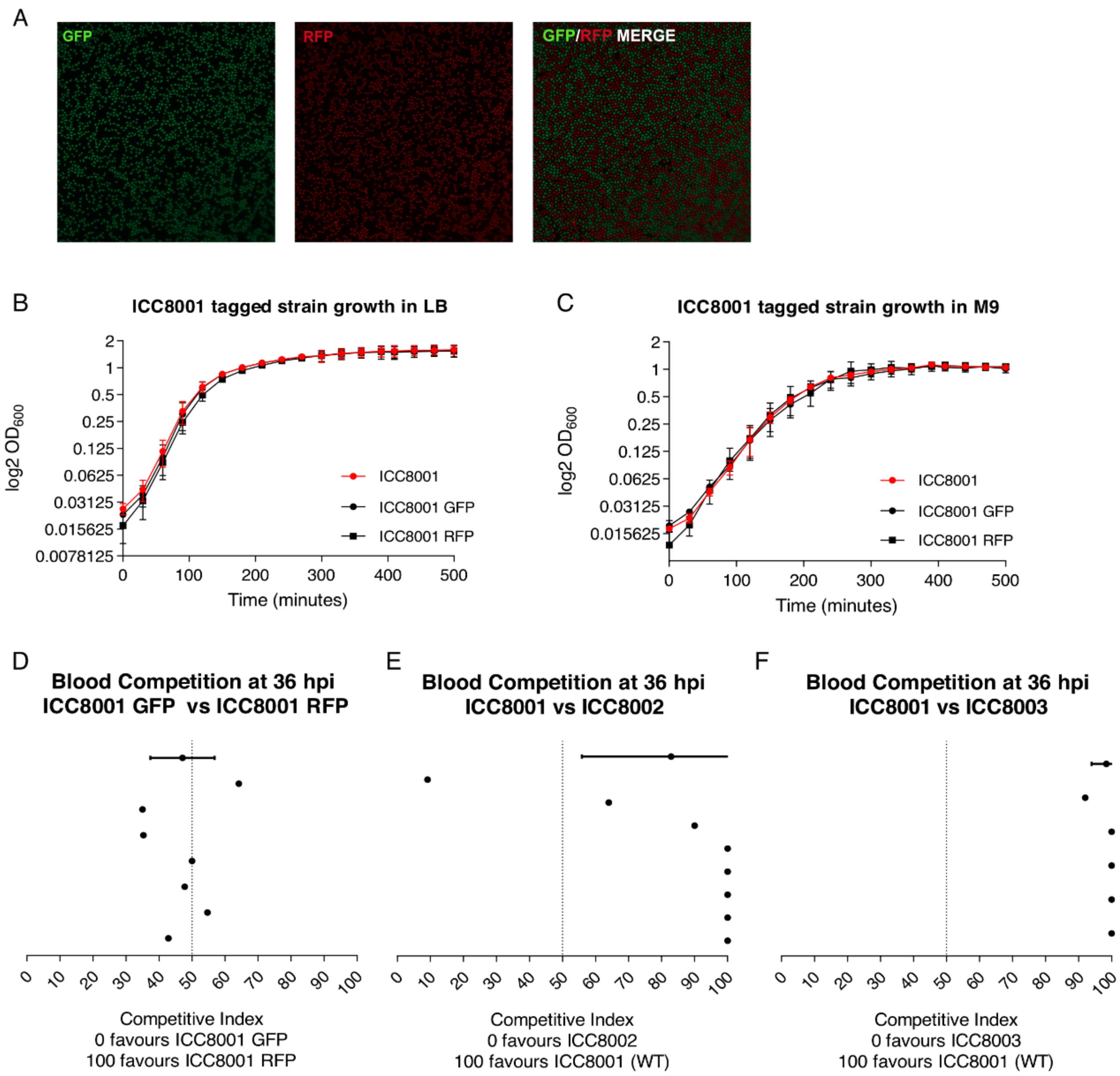

Supplementary Figure 9. Fluorescence-based *in vivo* competition assay and the impact of OmpK36$_{ST258}$ on dissemination from the lungs.

A. Expression of GFP and RFP was confirmed by fluorescence microscopy of mixed cultures (ICC8001GFP and ICC8001RFP) L-R single channel GFP/RFP and merge.

B. ICC8001 GFP and ICC8001 RFP demonstrate no apparent growth defect when compared to ICC8001 without a fluorescent tag in Luria Bertani or **C** M9 media (n=3 repeats, error bars=±s.d.).

D, **E, and F.** Competition in dissemination to the blood in animals from Main Figure 3 D, E. and F (n=10 infected IT per condition, error bars ±95% confidence interval).

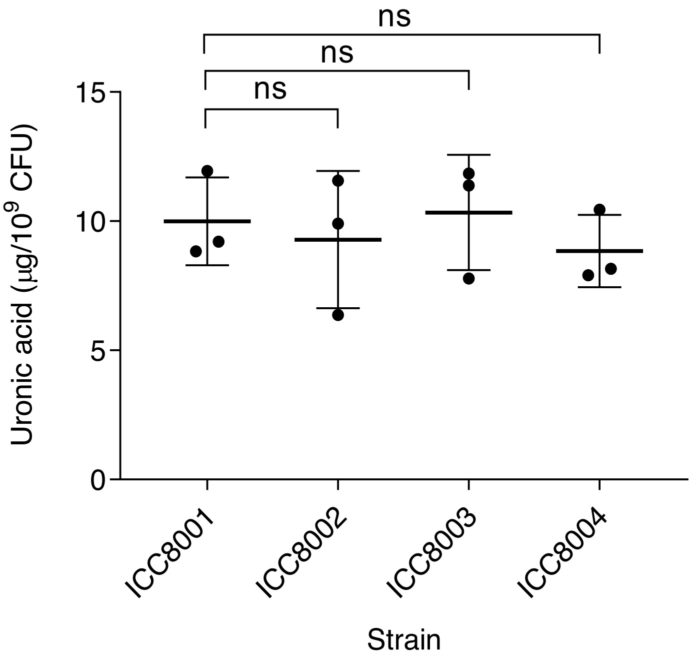

Supplementary Figure 10. Capsular polysaccharide abundance, assayed by measuring uronic acid, is not affected by ST258 porin substitution. (n=3 repeats, error bars=±s.d.)
