# Supplementary material for: OmpK36-mediated Carbapenem resistance attenuates ST258 Klebsiella pneumoniae in vivo
Source: Nat Commun. 2019 Sep 2;10:3957. doi: 10.1038/s41467-019-11756-y (PMC6718652; doi:10.1038/s41467-019-11756-y)
Supplement: Supplementary file 3 — Reporting Summary [file 41467_2019_11756_MOESM3_ESM.pdf]

nature research

Corresponding author(s): Gad Frankel

Last updated by author(s): 2019/07/23

Reporting Summary

Nature Research wishes to improve the reproducibility of the work that we publish. This form provides structure for consistency and transparency in reporting. For further information on Nature Research policies, see Authors & Referees and the Editorial Policy Checklist.

Statistics

For all statistical analyses, confirm that the following items are present in the figure legend, table legend, main text, or Methods section.

n/a	Confirmed	
☐	☒	The exact sample size (n) for each experimental group/condition, given as a discrete number and unit of measurement
☐	☒	A statement on whether measurements were taken from distinct samples or whether the same sample was measured repeatedly
☐	☒	The statistical test(s) used AND whether they are one- or two-sided<br>*Only common tests should be described solely by name; describe more complex techniques in the Methods section.*
☒	☐	A description of all covariates tested
☐	☒	A description of any assumptions or corrections, such as tests of normality and adjustment for multiple comparisons
☐	☒	A full description of the statistical parameters including central tendency (e.g. means) or other basic estimates (e.g. regression coefficient) AND variation (e.g. standard deviation) or associated estimates of uncertainty (e.g. confidence intervals)
☒	☐	For null hypothesis testing, the test statistic (e.g. F, t, r) with confidence intervals, effect sizes, degrees of freedom and P value noted<br>*Give P values as exact values whenever suitable.*
☒	☐	For Bayesian analysis, information on the choice of priors and Markov chain Monte Carlo settings
☒	☐	For hierarchical and complex designs, identification of the appropriate level for tests and full reporting of outcomes
☒	☐	Estimates of effect sizes (e.g. Cohen's d, Pearson's r), indicating how they were calculated

Our web collection on statistics for biologists contains articles on many of the points above.

Software and code

Policy information about availability of computer code

Data collection	Crystallographic data were collected at Diamond Light Source using the I03, I04 and I24 beamlines. GDA was used for the data collection (http://www.opengda.org)<br>In vivo imaging data acquisition was collected using the Living Image 4.5.5 software suite.
Data analysis	Crystallographic software: XDS, xia2, AUTOPROC, CCP4 package, Phenix, HOLE<br>In vivo imaging analysis took place in Living Image 4.5.5.<br>Plotting of data for graphs completed in Graphpad Prism for growth curves, pore radius, meropenem diffusion rates and infections in mice.<br>Non-linear regression, multiple comparisons ANOVA with corrections (described when each test applied in methods), calculations of confidence intervals were calculated in Graphpad Prism.

For manuscripts utilizing custom algorithms or software that are central to the research but not yet described in published literature, software must be made available to editors/reviewers. We strongly encourage code deposition in a community repository (e.g. GitHub). See the Nature Research guidelines for submitting code & software for further information.

Data

Policy information about availability of data

All manuscripts must include a data availability statement. This statement should provide the following information, where applicable:

- Accession codes, unique identifiers, or web links for publicly available datasets
- A list of figures that have associated raw data
- A description of any restrictions on data availability

The coordinates and structure factors of OmpK36WT, OmpK36ST258 and OmpK36WT+GD have been deposited to the Protein Data Bank with PDB ID codes 6RD3, 6RCP and 6RCK, respectively.

Field-specific reporting

Please select the one below that is the best fit for your research. If you are not sure, read the appropriate sections before making your selection.

☒ Life sciences ☐ Behavioural & social sciences ☐ Ecological, evolutionary & environmental sciences

For a reference copy of the document with all sections, see nature.com/documents/nr-reporting-summary-flat.pdf

Life sciences study design

All studies must disclose on these points even when the disclosure is negative.

Sample size	Sample size for animal work was calculated under the requirements stipulated in the United Kingdom Home Office License PPL 70/8413 granted by the Animals (Scientfic Procedures) Act 1986 (UK). For infections, this stipulates the use of 4-8 mice for all new infection conditions or new strains to obtain a 95% confidence interval using 2-tailed analysis of variance assuming uneven distribution. We employed 5 mice which represents the number of animals that can be co-housed in our current animal facility for each biological replicate.
Data exclusions	No data was excluded in the final analyses.
Replication	All infections in mice were replicated in biological duplicate (n=5 per replicate) for each condition tested. Replicates were temporally distinct providing parallel measurements of data (as outlined in https://www.nature.com/articles/nmeth.3091).<br>Liposomal swelling assays were conducted in triplicate with each biological repeat representing a distinct liposome reconstitution as outlined in the methods section. All growth curves and the assessment of capsule were conducted in biological triplicate.
Randomization	Mice were purchased from a registered supplier (Charles River, UK) and randomized into cages (n=5/cage) by staff of the Central Biomedical Services (Imperial College London) who are accredited by the Association for Assessment and Accreditation of Laboratory Animal Care (AAALAC). Central Biomedical Services provided animal husbandry but were not involved in the experimental design and provided cages of randomized co-housed animals to researchers carrying out the infections (authors JLCW and LEK).
Blinding	We were unable to blind researchers to the strains used to infect animals post inoculation as mice required extensive monitoring. Death is not an acceptable severe end point under the Animals (Scientfic Procedures) Act 1986 (UK) and the effect of porin substitution on virulence was unknown (before this manuscript). We complied with the legal framework by monitoring animal severity and keeping real time records of severity scores alongside the strain that had been administered. However, we only objective measures on infection at predefined time points (e.g. Lung CFU counts at 36 hour post infection) to remove any bias or influence of researchers knowing the strain that was being tested.

Reporting for specific materials, systems and methods

We require information from authors about some types of materials, experimental systems and methods used in many studies. Here, indicate whether each material, system or method listed is relevant to your study. If you are not sure if a list item applies to your research, read the appropriate section before selecting a response.

Materials & experimental systems

n/a	Involved in the study	
☒	☐	Antibodies
☒	☐	Eukaryotic cell lines
☒	☐	Palaeontology
☐	☒	Animals and other organisms
☒	☐	Human research participants
☒	☐	Clinical data

Methods

n/a	Involved in the study	
☒	☐	ChIP-seq
☒	☐	Flow cytometry
☒	☐	MRI-based neuroimaging

Animals and other organisms

Policy information about studies involving animals; ARRIVE guidelines recommended for reporting animal research

Laboratory animals	BALB/c females. 8-10 weeks 18-20g. No genetically modified animals were used.
Wild animals	None
Field-collected samples	None
Ethics oversight	The animal work was reviewed and approved by the Central Animal Welfare and Ethical Review Body at Imperial College London and approved under the Project license PPL70/8413 by the United Kingdom Home Office.

Note that full information on the approval of the study protocol must also be provided in the manuscript.
